# 

**Published:** 1873-10

**Authors:** 


					American Academy of Dental Science.?The sixth annual
meeting of this Academy was held in Boston, on Monday, Sep-
tember 29th. The annual address was delivered by Prof. P. H.
Austen, M. D., D. D. S., of the Baltimore College of Dental Sur-
gery, at 2 P. M.; and essays were read by Drs. N. "W. Kingsley,
of N. Y., Asa Hill, of Connecticut, and I. T. Codman, of Mass.
The annual dinner was served at the Parker House, at 5 P. M.
TO READERS AND C<>RRESRONDENTIS.
Communications intended for publication, and exchange journals and papersl
should be addresised to the Editor of the. American Journal of DentaiI
Science, 259 N. Eutaw St., Baltimore, Md. All letters relating to the business
of the i onrnal, or containing cash remittances, to be directed to Messrs. SnowdenI
& Cowman. Articles for publication should be received by the editor at least
three weeks previous to the first day of the month on which the number in which
it is designed for them to appear, is issued*
tSiTThe American Journal of Dental Science will contain fifty pae-es of
reading matter in each number, making a volume of not less than
Six Hundred pages
EXCLUSIVE OF ADVERTISEMENTS.
T H E
AMERICAN JOURNAL
OF
DENTAL SCIENCE.
F. J. S. GOKSAS, M. D., D, D. S.. Editor.
? The Journal is issued on the first of each month and contains not less
ihan fifty pages of reading matter in each number, exclusive of adver-
asements, inaking a volume of six hundred pages per annum.
*' In each number will be round Original Communications, Corres-
pondence, Selected Articles, Monthly Summary, Bibliographical No-
tices and Editorial Matter, and every effort will be exerted to render
the Journal worthy of the patronage of the Dental profession.
| A generous co-operation is respectfully solicited from the members
if the profession ; and as the Journal has now a printing office of its
own which materially lessens the cost of its publication, it will be
issued at the reduced subscription price of
$2.SO Per Annum, in iLdvance.
Communications are respectfully solicited on all subjects pertain-
ing to the practice of dentistry, as well as reports of cases occurring
in dental practice.
RATES OF ADVERTISING.
1 Page.
I h "
l\ "
1 MO.
$15 00
10 00
1 00
5 00
2 MO.
$22 50
15 00
11 00
8 00
3 MO.
$30 00
20 00
15 00
11 00
6 MO.
$50 00
30 00
20 00
15 00
12 MO.
$100 00
50 00
30 0C
20 00
| All original communications designed for publication, works for
review, new instruments for notice, exchanges, &c., should be directed
to the editor, 259 N. Eutaw St., Baltimore, Md.
All advertisements and subscriptions should be addressed to
SNOWDEN & COWMAN,
Publishers of the Am. Journal of Dental Science.
86 W. Fayette St. Bet. ChXrles & Liberty Sts.,
BALTIMORE, Md.

				

